# Characterization of the primary antibody response to *Plasmodium falciparum* antigens in infants living in a malaria-endemic area

**DOI:** 10.1186/s12936-022-04360-x

**Published:** 2022-11-19

**Authors:** Samuel Tassi Yunga, Chathura Siriwardhana, Genevieve G. Fouda, Naveen Bobbili, Grace Sama, John J. Chen, Rose F. G. Leke, Diane Wallace Taylor

**Affiliations:** 1 Department of Tropical Medicine, Medical Microbiology and Pharmacology, John A. Burns School of Medicine, University of Hawaii at Manoa, 651 Ilalo Street, Bioscience Building Suite 320, Honolulu, HI 96813 USA; 2 Department of Quantitative Health Sciences, John A. Burns School of Medicine, University of Hawaii at Manoa, 651 Ilalo Street, MEB411, Honolulu, HI 96813 USA; 3 Department of Pediatrics, Joan & Sanford I. Weill Medical College of Cornell University, New York, NY USA; 4 The Biotechnology Center, University of Yaoundé 1, Messa, BP 3851 Yaoundé, Cameroon; 5 Cancer Early Detection Advanced Research Center (CEDAR), School of Medicine, Knight Cancer Institute, Oregon Health & Science University, 2720 S. Moody Avenue, Portland, OR USA

**Keywords:** Malaria, *Plasmodium falciparum*, First antibody response, Babies, IgM, IgG

## Abstract

**Background:**

The primary antibody (Ab) response to *Plasmodium falciparum* is a critical step in developing immunity to malaria. Information on the initial Ab responses of babies in malaria-endemic areas is incomplete, in part, because babies receive maternal IgG via transplacental-transfer and usually become infected before maternal IgG wanes. The study aimed to identify the primary IgM and IgG Ab responses to malarial antigens in Cameroonian babies.

**Methods:**

Infants (n = 70) living in a high malaria transmission area were followed from birth throughout the first year of life (mean 341 ± 42 days, an average of 8.5 time points per infant). Malaria infection was assessed by microscopy and PCR, and IgM and IgG antibodies (Abs) were measured using a multiplex immunoassay to AMA1, EBA-175, MSP1-42, MSP2, MSP3, RESA, LSA1, and CSP.

**Results:**

The half-life of maternal IgG varied among the antigens, ranging from 0.7 to 2.5 months. The first infection of 41% of the babies was sub-microscopic and only 11 to 44% of the babies produced IgM to the above antigens; however, when the first infection was detected by microscopy, 59–82% of the infants made IgM Abs to the antigens. Infants were able to produce IgM even when maternal IgG was present, suggesting maternal Abs did not suppress the baby’s initial Ab response. Using longitudinal regression models that incorporated time-varying covariates, infants were found to produce IgG Ab to only AMA-1 when the first infection was sub-microscopic, but they produced IgG Abs to MSP1-42 (3D7, FVO), AMA1 (3D7, FVO) MSP2-FC27, MSP3, RESA, and LSA1, but not MSP 2-3D7, EBA-175, and CSP during their first slide-positive infection. Notably, the primary and secondary IgG responses were short-lived with little evidence of boosting.

**Conclusions:**

The primary Ab response of babies who had maternal IgG was similar to that reported for primary infections in malaria-naïve adults.

**Supplementary Information:**

The online version contains supplementary material available at 10.1186/s12936-022-04360-x.

## Background

Antibodies (Abs) play an important role in protection from falciparum malaria. The primary antibody (Ab) response is a critical step in the development of immunity as it ‘sets the stage’ for subsequent events in the maturation process. The primary anti-malarial IgG Ab responses of babies residing in high-transmission settings have been difficult to unravel for several reasons.

First, babies are born with anti-malarial IgG Abs acquired in utero from their mothers that wane during the first few months of life. Unfortunately, it is impossible to distinguish between maternal IgG and IgG produced by the baby. Longitudinal and cross-sectional studies have reported the decline of maternal IgG in groups of infants and the subsequent increase in IgG Ab as babies become infected [1–12]. Antigens (Ags) that have been studied include exoerythrocytic-stage antigens (CSP, LSA1, TRAP) [3, 4, 6, 11], merozoite antigens (MSP1, MSP2, MSP3, AMA1, EBA-175 [3, 5, 6, 8, 10–12], as well as PfEMP1, RESA, GLURP, and PfSEA-1 [2, 4, 7–9]. These data provide a generalized picture of the change in IgG for these malaria-specific antigens, but they do not fully characterize the primary response in individual babies. Thus, many questions remain: (i) about the level of parasite burden needed to stimulate an IgG response; (ii) if the neonatal immune system can recognize malarial antigens in the presence of maternal IgG (i.e., do maternal Abs inhibit the neonatal response); (iii) if class-switching from IgM to IgG occurs during primary infection; and, iv) if Abs are produced against all malarial antigens or if the initial response is restricted to immunodominant antigens or epitopes.

Secondly, primary *Plasmodium falciparum* infections in babies may be asymptomatic with very low or sub-microscopic infections [13] that are transient and cleared without treatment [14, 15]. It is currently unclear if these early, sub-patent infections are adequate to stimulate an Ab response. Previous studies have examined the Ab response during the first clinical episode of malaria; however, babies in endemic areas rarely develop clinical disease before 5–6 months of age [14–17]. Thus, the Ab response associated with a clinical infection may not be the primary response, but rather reflect the summation of prior exposures. Third, prior studies that measured Abs to one or a few antigens provide information about specific antigen(s), but they do not give an overview of the response to antigens with different immunogenicities (e.g., immunodominant *vs* cryptic). Finally, first infections may be missed if blood samples are not collected frequently or assessed by PCR. Surprisingly, only a few studies have used PCR to detect parasitaemia in infants during the first year of life [13, 18–20].

The current study sought to characterize the primary Ab response of 70 newborns residing in the rural village of Ngali II, Cameroon, where *P. falciparum* transmission is perennial and individuals receive ~ 257 infectious mosquito bites annually [21]. Infections were detected by PCR and microscopy, and IgM and IgG Abs to 8 *P. falciparum* antigens were measured. The dataset was used to help answer relevant questions, including: (i) do early sub-microscopic infections induce IgM and IgG Ab to the 8 antigens or is parasitaemia detected by microscopy required to induce a response?; (ii) do babies produce both IgM and IgG Abs upon primary infection?; (iii) do infants produce IgM and/or IgG Ab in the presence of maternal IgG Abs?; and, (iv) is the Ab repertoire produced during the initial response the same as that produced during the first clinical malaria episode? Overall, the dataset helped fill some of the gaps in our knowledge about the primary anti-*P. falciparum* Ab response of infants residing in high transmission settings.

## Methods

### Study design

The study was performed as previously described in the rural community of Ngali II, near Yaoundé, Cameroon, where individuals receive an estimated 257 infectious mosquito bites annually [19, 21]. From May 2001 to November 2004, pregnant women were informed about the project and the newborns of women who signed informed consent forms were included as study participants. At enrolment, delivery information was recorded and maternal venous and placental blood samples were collected. After birth, infants were monitored regularly during programmed visits at 9 time points: 7 days, 6 weeks, 3, 4, 5, 6, 8, 10 and 12 months of life [19]. At each visit, infant peripheral blood samples were collected by finger prick and used for parasitological and Ab studies. Samples collected from 70 infants who were not lost from follow-up before the 8th-month visit were included in the current study.

### Parasitological studies

Thick- and thin-blood smears of maternal venous and baby finger prick blood were prepared, stained with Diff-Quick, and examined for parasites by microscopy. Total WBC/µl were determined from the corresponding blood samples and results were calculated as number of infected erythrocytes (IE)/µl of blood. In addition, placental intervillous space (IVS) blood samples were collected and examined for parasites by microscopy, as well as, histological sections of placental biopsies, stored in 10% buffered formalin, were prepared, stained with Hematoxylin–Eosin and examination by microscopy. Placental malaria (PM) was defined as the presence of *P. falciparum* IE in IVS blood smears and/or in histological sections.

### Detection of infection by PCR

*Plasmodium falciparum* infections were also detected in peripheral blood samples of infants by PCR during the first year of life. PCR was performed by amplifying the 18S ribosomal subunit gene of *P. falciparum* using primers and protocols that have been described previously [22, 23].

### Antibody studies

Abs to 8 *P. falciparum* antigens were measured, including 5 *P. falciparum* merozoite-stage Ags (MSP1 [3D7, FVO], MSP2 [FC27], MSP3, AMA1 [3D7], EBA175), one ring-stage erythrocyte surface Ag (RESA), and 2 exo-erythrocytic-stage Ags (CSP and LSA1) using a multiplex Luminex assay. The description of each Ag, including the amount of Ag used for coupling, is provided in Additional file 7: Table S1. A mixture of beads was prepared, containing 100 beads/µl of each antigen coupled to a different spectral address, and 50 µl of the mixture in PBS-1% BSA was combined with 50 µl of a 1:100 dilution of plasma (total dilution 1:200) in filter plate wells (Multiscreen BV, Millipore). After one hour of incubation in the dark on a 500-rpm shaker at RT, the wells were washed 5 times with PBS-0.05% Tween 20. Then, 100 µl of secondary Ab, either 0.1 mg of R-phycoerythrin conjugated to the F(ab’) 2 fragment of goat anti-human IgG or to the F(ab’) 2 fragment of donkey anti-human IgM (Jackson Immunoresearch) was added to each well. After 30 min of incubation, the plates were washed again 5 times and the beads were resuspended in 100 μl PBS-1% BSA. The plates were then read in the Liquichip M100 reader (Qiagen) that detected the spectral address of each bead type (hence corresponding Ag) and the median fluorescence intensity (MFI) of phycoerythrin (i.e., amount of Ab bound). Pooled positive and negative control plasma samples were included on each plate. The cut-off for waning maternal IgG Ab was the mean + 3 SD of the lowest MFI each baby had prior to a rise due to Ab production by the baby. The cut-off for IgM Abs was the mean MFI ± 3SD at the first visit (mean 9 ± 2 days), after deleting outliers that were 1 SD above the combined mean. These cut-off values were selected rather than the traditional MFI from naïve adult controls (e.g., plasma from Americans), since these cut-offs reflect MFI in young babies in rural setting who were becoming infected with a variety of pathogens.

### Statistics

Results from the 70 infants were summarized using descriptive statistics, either as means ± standard deviations (SD) or median ± interquartile range (IQR) based on distribution of the data. The rate of antibody decline was determined using a longitudinal linear mixed-effects model. The model accounts for repeated measurements from an infant and it was used with the log-transformed values of the antibody observed at multiple time points. The half-life estimate (${T}_{1/2}$) was calculated using the equation: ${T}_{1/2}=\mathrm{ln}(2)/\beta ,$ where $\beta$ is the estimated slope parameter of the linear mixed-effects model [24–26]. The half-life estimation was performed using data from the first 6 months of life, and provided with 95% confidence intervals (CI), for a set of Abs observed to be decreasing over time. Next, statistical tests were conducted to determine if *P. falciparum* IgG and/or IgM levels in infants were significantly increased at first *P. falciparum* infection or if PM status influenced the result. A set of linear mixed effect models were generated considering the responses as log-transformed Ab levels tracked from day 7 of life to the time of first *P. falciparum* infection or to the last follow-up visit if no infection occurred. First *P. falciparum* infection was the predictor of primary interest. Adjustments were made for covariates, such as baseline Ab level, length of time from baseline to infection, and for grouping variables such as PM status. Variable associations were determined by testing regression coefficients based on the Wald test. In mixed effect models applied, subject-specific intercepts were used as random components. All statistical analyses were performed using R software version 4.0.2 or GraphPad Prism 9.

## Results

### Characteristics of the infants

The 70 infants enrolled in the study were healthy newborns, with an average gestational age of 39.2 ± 2.0 weeks and weighing 3,177 ± 484 g (Table 1). Only 7.9% of the babies were low birth weight (LBW), although 46.6% of the mothers were PM-positive (PM +) at delivery. The babies were followed longitudinally through the first year of life, with a mean follow-up time of 341 ± 42 days. Babies were seen an average of 8.5 times (range 6–9) during the first year of life.

**Table 1:** Description of infants residing in Ngali II

Number of infants	70
Gravidity (median, range)	3 (range 1–12)
Gestational age at birth (weeks)^a^	39.2 ± 2.0
Percent full-term births	89.9%
Birth weight (grams)^a^	3,177 ± 484
Percent low-birth weight (< 2,500 g)	7.9%
% of women with placental malaria (PM-pos)	46.6% (27/58)
Mean number of visits per infant	8.5 (range 6–9)
Duration of follow-up (days from birth)^a^	341 ± 42
Mean number of times malaria-positive by microscopy (range)	2.7 ± 2.1 (range: 0 to 7)
Mean number of times malaria-positive by PCR (range)	3.9 ± 2.1 (0 to 8)

^a^Mean ± SD

### Plasmodium falciparum infections during the first year of life

**Fig. 1:**
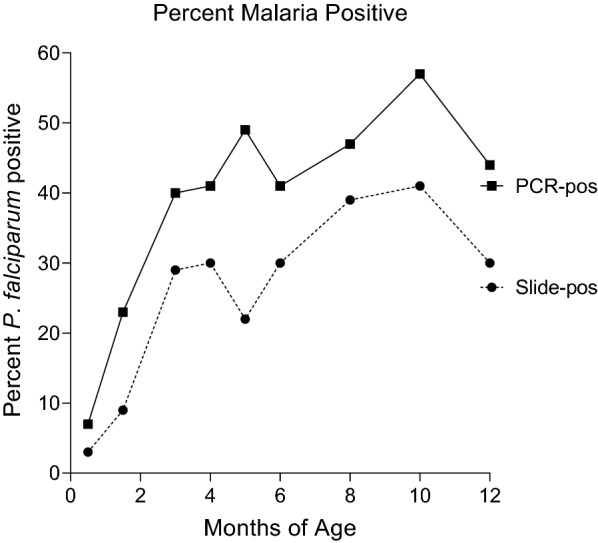
Infants positive for *Plasmodium falciparum* by PCR and microscopy at different ages (n = 70)

At the first time point (mean 9 ± 2 days), only 4% of the newborns were malaria-positive by microscopy and 7% by PCR, suggesting few congenital infections (Fig. 1). The number of infants infected with *P. falciparum* increased rapidly over the first 4 months of life and then remained relatively constant thereafter. Overall, *P. falciparum* infections were detected in infants 2.7 ± 2.1 (range 0 to 7) and 3.9 ± 2.1 (range 0 to 8) times by microscopy and PCR, respectively, during the year (Table 1).

### Time until the first infection

**Fig. 2:**
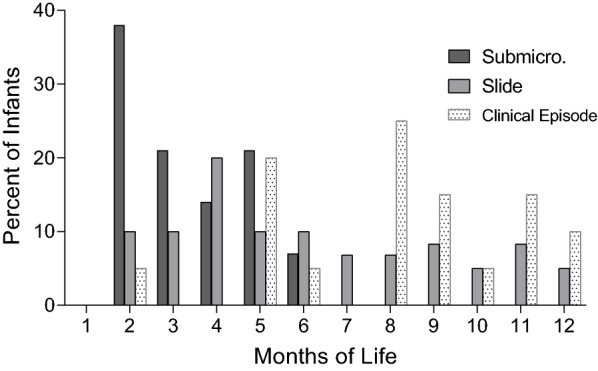
Time of the first *Plasmodium falciparum* infections and clinical episode. The first infection of 29 babies was a sub-microscopic infection. Among the 70 infants, 60 developed infections detected by microscopy at the time points shown. Only 18 infants had symptomatic clinical episodes of malaria, mainly after 5 months of age

Longitudinal data identified when each baby had his/her first *P. falciparum* infection that was either submicroscopic (i.e., PCR-positive, slide-negative) or detected by microscopy, and symptomatic episode of malaria (Fig. 2). Overall, first infections of 29 babies were sub-microscopic and occurred between the 2nd and 6th month of life. Among these 29 babies, 83% (24/29) were malaria-negative at the next time point (~ 1 month later) by microscopy, showing these babies initial infections were transient and self-resolving with very low parasitaemia. On the other hand, the first microscopically detected infection occurred in 86% (60/70) of the babies, (including some babies who had prior sub-microscopic infections) between 2 to 12 months of life (median of 6 months). Overall, 14% of the babies were malaria-negative by PCR and microscopy throughout the first year of life. Only 26% (18/70) of the babies developed clinical cases of malaria (defined as slide-positive for *P. falciparum* with axillary temperature ≥ 37.5 °C), that were not severe, with the first episode usually occurring after 5 months of age.

### Decline of maternal IgG

**Fig. 3:**
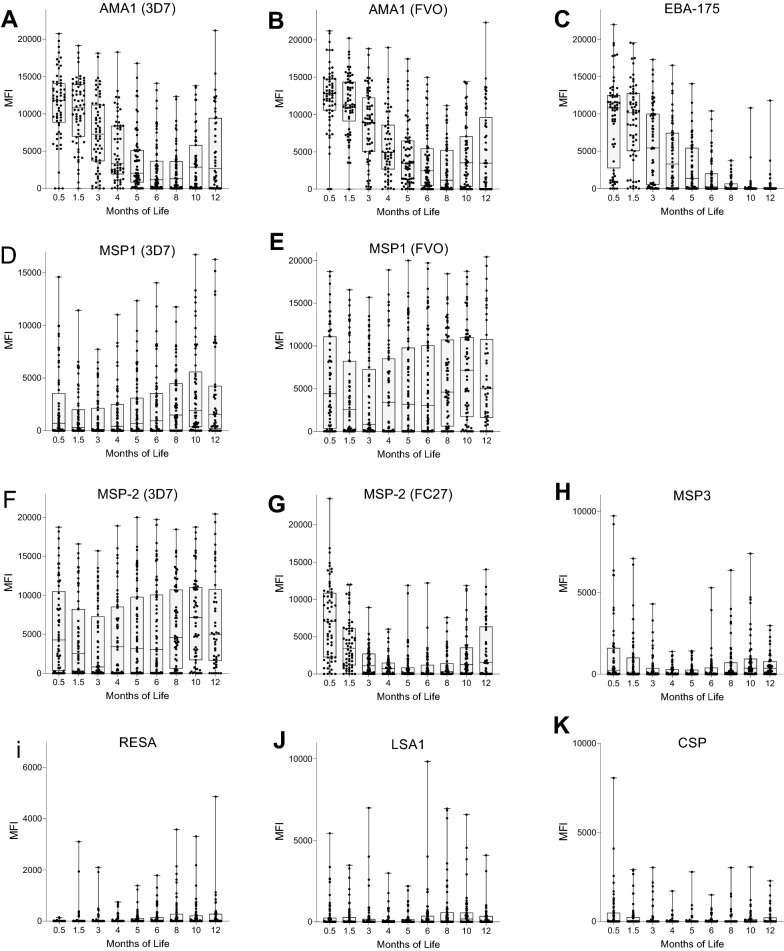
Changes in IgG antibody levels during the first year of life IgG. Antibody levels were measured in the 70 infants during the first year of life at 9 time points. A. AMA-1 (3D7), B. AMA1 (FVO); C. EBA-175; D. MSP1-42 (3D7); E. MSP1-42 (FVO); F. MSP2(3D7); G. MSP-2 (FC27); H. MSP3; I. RESA, J. LSA1, and K. CSP. Since MFIs were not normally distributed, scattergrams are presented as Boxplots showing medians and interquartile ranges

The decline of maternal IgG Ab to 8 malarial antigens (11 recombinant proteins) during the first year of life is shown in Table 2 and Fig. 3. At ~ 1 week of age, babies had high (> 10,000 MFI) levels of maternal IgG Ab to AMA1, EBA-175, (Fig. 3A–C); intermediate (> 1,000 but < 10,000 MFI) Ab levels to MSP1-42 (3D7, FVO) and MSP2 (3D7, FC27) (Fig. 3D-G), and very low levels (< 1000 MFI) to MSP3, RESA, LSA-1 and CSP (Fig. 3H–K). Overall, 80–97% of newborns had maternal IgG Ab to AMA1 (3D7, FVO) and EBA-175 that were detectable for 6 to 8 months, with Ab half-lives ranging from 0.98 to 1.9 months based on data for the first 6 months of life (Table 2). On the other hand, Abs to MSP1-42 and MSP2 declined quickly to background (cut-off) levels by 3 months of age. Thereafter, IgG Ab levels to MSP1-42 and MSP2 quickly increased as the babies produced Abs when they became infected with *P. falciparum*. Ab half-life estimates for the first 6 months of life was 0.7 months for MSP2, but the half-life to MSP1-42 could not be determined over this period since infants produced IgG to MSP1-42 as early as 3–4 months of age. On the other hand, most infants had very low or no Ab levels to MSP3, RESA, LSA1, and CSP (Fig. 3H–K). As a result, a decrease in maternal IgG over time was not observed, preventing an estimate of Ab half-live.

**Table 2:** Characteristics of maternal IgG transplacentally transferred to babies

	Amount of Ab MFI at day 9 ± 2 (median, 1st, 3rd quartile)	Percentage of Ab-positive Babies^a^	Ab Half-life, ± 95% CI (months)^b^	Length of detectable maternal IgG (months)
AMA1 (3D7)	11,757 (8,765, 14,108)	96.9	1.46 (1.17, 1.94)	6
AMA1 (FVO)	12,581 (10,218, 14,870)	96.9	1.88 (1.4, 2.59)	8
EBA-175	10,800 (3,635, 12,460)	79.7	0.98 (0.83,1.21)	6
MSP1-42 (3D7)	1,003 (10, 3,868)	35.9	NC^c^	NC
MSP1-42 (FVO)	4,637 (615, 11,207)	67.2	NC	3
MSP2 (3D7)	6,235 (2,298, 10,163)	76.6	0.78 (0.67, 0.92)	3
MSP2 (FC27)	6,935 (2,325, 10,163)	81.3	0.73 (0.64, 0.86)	NC
MSP3	239 (0, 1,482)	31.3	2.41 (1.58, 5.04)	NC
RESA	0 (0, 0)	0	NC	NC
LSA-1	0 (0, 218)	23.4	NC	NC
CSP	0 (0, 352)	25.0	2.39 (1.64, 4.36)	NC

^a^Percent of babies at 9 ± 2 days with MFI above cut-off: AMA1(3D7) 1,970 MFI; AMA1 (FVO) 2,050 MFI; EBA-175 (2,020 MFI; MSP1(3D7) 1,500MFI; MSP1(FVO) 1,800 MFI; MSP2(3D7) 1,980 MFI; MSP2(FC27) 1,880 MFI; MSP3 842 MFI; RESA 390 MFI; LSA-1 380 MFI; CSP 490 MFI

^b^based on IgG Ab levels between Day 9 ± 2 after birth until 6 months of age

^c^Not calculatable, because no consistent decline occurred or Ab were produced prior to 6 months

### Relationship between maternal IgG levels at birth and primary parasitaemia

Ab levels at the first visit were compared between babies whose first infections were sub-microscopic (n = 18), i.e., very low parasitaemia, and those with slide-positive infections (n = 52, range 21–150,000 IE/µl), i.e., higher parasitaemia. No difference in median MFI was found (all p values > 0.05), except for one antigen where MFI were higher in the slide-positive group (Additional file 1: Fig. S1). Thus, the data suggest that high maternal IgG levels to any of the 8 antigens at birth did not help reduce parasitaemia to sub-microscopic levels.

### The IgM antibody response of infants during the first year of life

IgM Ab data were available for 47 babies who had ≥ 7 visits (Additional file 2: Fig. S2). The results allowed an answer to the following three questions:

Is the parasitaemia high enough during the first sub-microscopic infection to induce an IgM Ab response? The first infection was sub-microscopic in 38% (18/47) of these babies. At that time, relatively few babies had IgM Ab, with i) 35–44% of babies having IgM Abs to AMA-1 (3D7); ii) 27–33% having Ab to AMA-1 (FVO), MSP-1 (FVO), EBA-175, MSP-2 (3D7, FC27); and, iii) only 11–22% having Abs to RESA, MSP3, LSA-1, and CSP (Table 3). Since the majority of babies were slide-negative for *P. falciparum* at the next visit (~ 1 month later), these early transient sub-microscopic infections induced an IgM response in only a small proportion of infants. On the other hand, when the first infection was high enough to be detected by microscopy, 68–82% of the babies had IgM to the 8 antigens (Table 3). Thus, sub-microscopic infections could induce IgM in some babies, most babies produced IgM to multiple antigens only when they had parasitaemia detectable by microscopy.

**Table 3:** Percentage of infants positive for IgM antibodies at their first sub-microscopic and slide-positive infections, as well as at the first symptomatic clinical episode

Antigens	Sub-microscopic infection^a^ (n = 18)	Slide positive infections^b^ (n = 39)	First clinical episode (n = 11)
AMA-1 (3D7)	44.4	76.9	73
AMA-1 (FV0)	33.3	82.1	91
EBA-175	27.8	66.7	73
MSP-1 (3D7)	22.2	76.9	91
MSP-1 (FVO)	33.3	79.5	91
MSP-2 (3D7)	27.8	79.5	91
MSP2 (FC27)	27.8	74.4	82
MSP-3	11.1	59.0	91
RESA	11.1	71.8	64
LSA-1	16.7	74.4	91
CSP	22.2	69.2	82

^a^PCR-positive, but slide negative

^b^First slide-positive infection

Did the infants produce IgM Ab to all 8 malarial antigens or is the response more restricted? As shown in Table 3, babies with primary sub-microscopic infections responded to a restricted repertoire of antigens, showing that some antigens were more immunogenic than others, e.g., 44% of babies with sub-microscopic infections produced IgM to AMA1; whereas, only 11% produced IgM to RESA and MSP3. On the other hand, most babies had IgM Abs to most (i.e., 59 to 82%) of the 8 antigens at the time of their first slide-positive infection (Table 3). Although most babies had Abs to many antigens, variation occurred among the babies, e.g., one baby might have Abs to AMA1, but lack Abs to RESA; whereas, another baby would have Abs to MSP1, but not EBA-175. At the time of the first symptomatic malaria episode, which usually occurred after infants had had several asymptomatic infections, 64 to 91% of infants had IgM Abs to the 8 malarial antigens (Table 3). Thus, following the first sub-microscopic infection the response was quite restricted, but when parasitaemia was high enough to be detected by microscopy most babies produced IgM to most of the antigens studied.

**Fig. 4:**
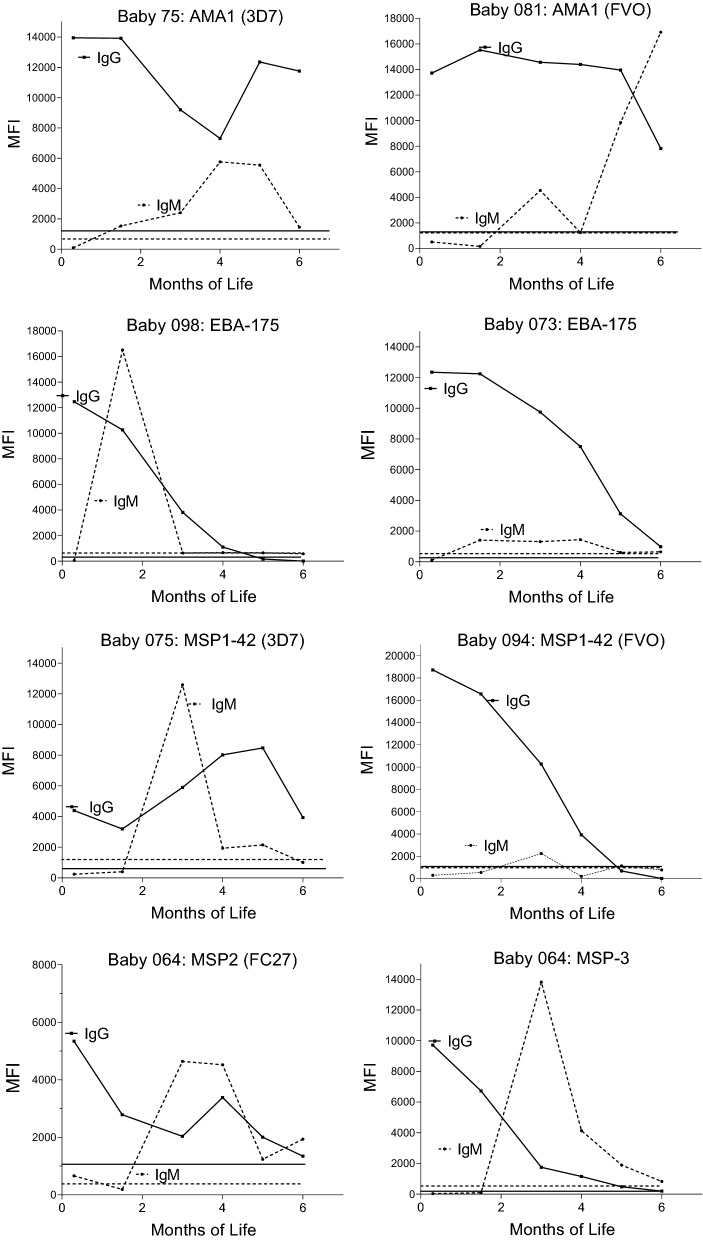
Comparison of declining maternal IgG with the baby’s IgM response. Each graph represents a single baby and one malarial antigen. The horizontal lines at the bottom show the cut-off for positivity: dotted lines (- - - -) for IgM and solid (____) lines for IgG Abs

Do infants produce IgM Abs before maternal IgG levels wane, i.e., reach background levels? To answer this question, the primary IgM response (i.e., the first visit when the MFI was above the cut-off) was compared with the corresponding maternal IgG level to determine if the MFI was still positive (above cut-off). Although the first IgM response often occurred in babies who either had not received maternal IgG for the antigen or after maternal Ab had waned (dropped below the IgG cut-off), a significant proportion of infants produced their first IgM response while significant amounts of maternal IgG were still present (Table 4). For example, over half the babies had maternal IgG to MSP1, 60.9% to EBA-175, and > 76% had IgG to AMA1 at the time they first produced IgM to these antigens (Table 4). Examples of the production of infant IgM in the presence of maternal IgG are shown in Fig. 4. Thus, babies can produce a humoral response to antigens even when maternal IgG levels remain positive.

**Table 4:** Percentage of infants who produced an IgM response in the presence of maternal IgG

Antigens	AMA-1 3D7	AMA-1 FVO	EBA-175	MSP-1 3D7	MSP-1 FVO	MSP 2 FC29	MSP3
	27.3% (12/44)	34.1% (15/44)	60.9% (28/46)	77.8% (35/44)	76.2% (35/46)	36.6% (15/41)	13.3% (6/45)

Results for RESA, LSA1 and CSP are not included because Ab levels to these antigens were very low and no sustained downward trend in Ab levels was observed over the first few months of life

### The IgG antibody response at first post-natal infection

Because of declining maternal IgG Abs in young babies, it is often difficult to determine when a baby first produces IgG Abs after infection (i.e., a rise in IgG Abs). Accordingly, longitudinal regression models were created that took the decline of maternal IgG into consideration when assessing if a baby produced IgG upon primary i) sub-microscopic infection; ii) slide-positive infection; and, iii) when the infant had its first clinical episode of malaria (Table 5). Data included in the analysis were from all 70 infants. Positive regressing results are shown in Table 5, where coefficients with significant p values indicate that an IgG response had occurred. Among the babies with primary sub-microscopic infections, IgG Abs were only produced to AMA1 (3D7, FVO). However, when parasitaemia were adequate to be detected by microscopy, IgG Abs to 5 antigens were produced, including MSP1 (3D7, FVO), MSP2 (FC27), AMA1 (3D7, FVO), RESA and LSA1, but not to MSP2 (3D7), EBA-175, and CSP. A similar pattern was found in the infants who developed clinical episodes of malaria later in life (Table 5). Taken together, data show that upon initial sub-microscopic infections, babies produced IgG Ab to AMA1; however, when parasitaemia reached detectable levels by microcopy IgG Ab to most antigens were detected.

**Table 5:** IgG response upon primary *Plasmodium falciparum* infection (Coefficient, p-value)

	Sub-microscopic^a^	Slide-positive	Clinical episode
AMA1(3D7)	0.81; p = 0.036	1.17; p = 0.004	
AMA1 (FVO)	0.97; p = 0.019	1.00; p = 0.008	1.73; p = 0.026
EBA-175			
MSP1 (3D7)		2.30; p < 0.0001	2.14; p = 0.033
MSP1 (FVO)		2.04; p = 0.001	
MSP2 (3D7)			4.01; p < 0.001
MSP2 (FC27)		0.97; p = 0.047	3.26; p < 0.001
MSP3		1.23; p = 0.011	1.65; p = 0.048
RESA		1.31; p < 0.001	2.42; p = 0.001
LSA1		1.28; p = 0.003	1.76; p = 0.023
CSP			1.82; p = 0.007

^a^Excluded all babies whose first infection was microscopically-positive and PCR-negative and microscopic-positive and PCR-positive

BLANK = negative coefficient value or non-significant p value

### IgM and IgG Ab responses were short-lived

**Fig. 5:**
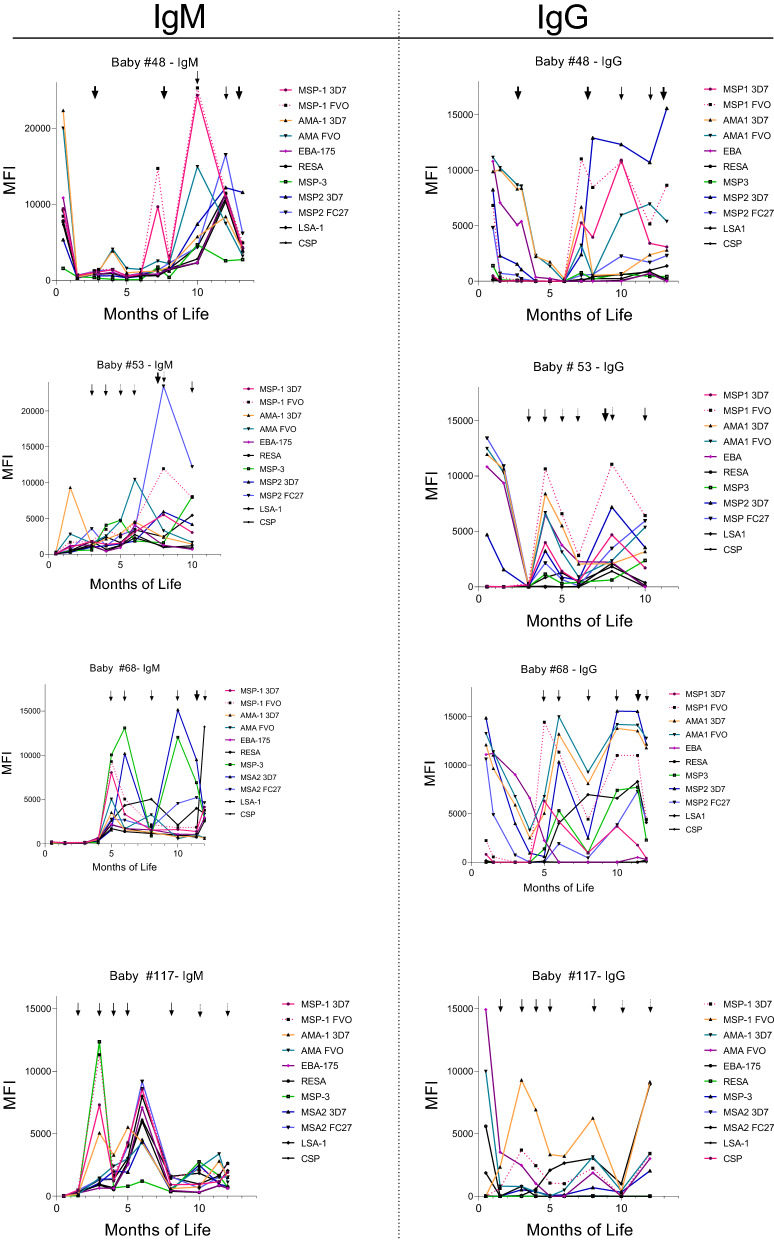
Examples of the IgM and IgG levels in four infants during the first year of life. The arrows indicate when malarial parasites were detected by PCR (dotted arrows), slide (solid arrows), and when a clinical episode occurred (bold arrows)

During the first year of life, IgM and corresponding IgG levels increased one or more times and then quickly declined following recent infection (Fig. 5, Additional file 3: Fig. S3). Some babies had second and third peaks of IgM and IgG, which also declined quickly (Fig. 5). In general, primary and secondary IgG responses were similar with minimal or no increase in Ab levels or breadth of the response (Additional file 3: Fig. S3). Overall, a true primary versus secondary response with higher IgG levels and broader antigenic specificity was not observed in infants during the first year of life (Fig. 5, Additional file 1: Fig. S3).

### Ab levels at birth were not associated with time to first infection or number of infections

No association was found between Ab levels to any of the antigens at week one of life (i.e., the first time point (mean 9 ± 2 days) and i) time to first infection, ii) number of infections detected by PCR or iii) number of infections detected by microscopy during the first year of life (Additional file 4: Fig. S4, Additional file 5: Fig. S5, additional file 6: Fig. S6).

## Discussion

The primary Ab response to *P. falciparum* is important in establishing the initial repertoire of activated B cells, induction of class-switching, and development of plasma and memory B cells. Previous studies have monitored the primary Ab response in malaria-naïve travelers who contacted malaria abroad and in transmigrants upon moving into a malaria-endemic area [27–34]. Studies show that IgG Abs are rapidly produced during illness to multiple sexual and asexual-stage antigens [27, 29–31, 33–35]; variation in the amount and breadth of the Ab response occurs among individuals; and primary Ab levels are lower than those produced upon secondary infection [28, 30, 33–35]. The primary IgG Ab response to *P. falciparum* is short-lived [29, 30, 32, 34], with half of individuals becoming Ab-negative in 1.6 to 31 weeks depending on the antigen [30]. The influence of age on the primary Ab response is currently being debated, since similar Ab responses in naïve Mozambican children and European adults were found during their primary infections [27]; however, a study of Javanese transmigrants found that the humoral response was age-related [34]. The current study sought to determine if a similar pattern of immune-responsiveness took place in babies during the first year of life who had circulating maternal IgG, since anti-malaria IgG might complex with antigens and prevent immune activation.

At birth, maternal IgG was detected in all newborns, but substantial variation was observed among the babies, with respect to (i) the antigen-specificity (repertoire) of maternal Abs (Table 2); (ii) the amount of maternal IgG (Fig. 2); and, (iii) the rate of decline (half-life) of maternal IgG for the various antigens (Table 2). In general, at one week of age, newborns were likely to have maternal IgG Abs to AMA1, EBA-175, MSP1 and MSP3 (Table 2), but little or no Abs to MSP3, RESA, LSA-I and CSP (Fig. 3). Thereafter, maternal Abs declined with Ab half-lives ranging from 0.7 to 2.4 months (i.e., mean 21 to 75 days) depending on the antigen (Table 2). Although immunological text books state that the half-life of human IgG is ~ 3 weeks (21 days), a wide-range of Ab half-lives has been reported for passively transferred IgG. The half-life of IgG is dependent on i) the starting Ab concentration (e.g., an inverse relationship between Ab levels and Ab half-lives has been reported) [24]; ii) the total amount of IgG present in circulation; and, iii) relative saturation of Fc neonatal receptors (nFcR). For example, the half-life of passive transfer replacement IgG was found to be 22 to 96 days [36] and the half-lives of maternal IgG in newborns was 29 days to pertussis [37], 40 days to hepatitis A virus [38], 79 days for respiratory syncytial virus [39], and 75 to 147 days (2.5 to 4.9 months) for *P. falciparum* merozoite antigens in Kenyan infants [24]. Thus, although maternal Ab levels are generally reported to wane in 2–6 months [3, 10–12], the half-life for each antigen is highly variable.

The contribution of maternal IgG to ‘protection’ of newborns is unclear, as some studies have found no relationship between that amount of maternal IgG at birth and time to first infection or risk of infection during the first year of life (reviewed in [15]). In fact, some studies have even reported that higher levels of anti-malarial Ab in newborns at birth were associated with an increased risk of infection [20, 40]. On the other hand, other studies have found a potential protective role for Ab to PfSEA-1 [9], endothelial protein C receptor-binding CIDRα1 [2, 41], and possibly MSP1-19 [18, 42, 43]. It is tempting to hypothesize that maternal IgG Ab helps reduce parasitaemia to low or sub-microscopic levels during the first few months of life [4]; however, data supporting the hypothesis are lacking. A comparison of Ab levels during the first week of life in babies having either i) a sub-microscopic parasitaemia, or, ii) a parasitaemia high enough to be detected by microscopy did not find a relationship between high Ab levels at birth and parasitaemia at first infection (Additional file 2: Fig. S2). The data were also analysed in multiple ways, but no evidence was found that Ab levels to any of the 8 individual antigens studied, including MSP1-42, were associated with fewer infections or shorter time to first infection detected by PCR or microscopy (Additional file 4: Fig. S4, Additional file 5: Fig. S5, additional file 6: Fig. S6). Clearly, immunity is mediated by Abs to a combination of multiple antigens, but there was no evidence in this study that Abs to any of the 8 antigens alone played a role in the control of asymptomatic, self-limiting submicroscopic infections early in life (Table 3).

Throughout the first year, essentially all babies had one or more sub-microscopic and/or slide-positive infections; whereas, only a few infants developed clinical infections (Fig. 1, Table 3). Although the infants were carefully monitored, seroconversion was occasionally detected in the absence of parasitaemia, demonstrating that not all infections had been documented. Thus, the data in Fig. 1 are an underestimate of the true prevalence of malaria in the village. Most sub-microscopic infections were identified during the second and third months of life (Fig. 2). The question became, did these early infections stimulate a humoral response? Results showed that during early sub-microscopic infections only 11 to 44% of the babies had produced IgM Abs to the 8 antigens, and only IgG Abs to AMA-1 (Tables 3 and 5). However, during the first slide-positive infection, more than half of the babies (i.e., 59 to 82% depending on the antigen) produced IgM and modelling revealed a significant increase in IgG to all of the antigens except EBA-175, MSP2 (3D7) and CSP (Tables 5 and 3). Since the lower limit of detection of *P. falciparum* by thick-blood film is ~ 100 IE per µl and that of the PCR test used in this study was ~ 1 IE/µl; the results suggest that parasitaemia of > 100 IE/µl may be needed to induce an Ab response in most babies, especially to weaker antigens. Currently, information on the amount of antigen or parasitaemia needed to induce an anti-malarial humoral response in infants is unknown.

Although maternal IgG was still detectable in infant circulation, about one-third of babies produced IgM Ab to AMA1, MSP2, and MSP3 and over 50% of babies made IgM to MSP1-42 and EBA-175 during their primary sub-microscopic or slide-positive infections (Table 4, Fig. 4). Previously, it was unclear if passively acquired maternal IgG would complex with malarial antigens and deplete their concentrations to sub-immunogenic levels; therefore, preventing the generation of plasma B cells. However, such does not appear to be the case. Similar results were recently reported by Park et al. showing that maternal Abs in cord blood did not abrogate the development of an infant’s Ab response to the *P. falciparum* schizont egress antigen-1 (PfSEA-1) [9]. These results suggest that either maternal vaccination or a vaccine given soon after birth may be feasible for protecting young babies from severe disease.

One problem in studying the primary IgG response in babies is that it is not possible to distinguish between IgG made by the babies, which may be produced in small amounts, from maternal IgG, that may be present in relatively high concentrations. Accordingly, a statistical approach was used that took into consideration variables that influence the primary Ab response, including the decline of maternal IgG (Table 5). Among the 70 babies, only IgG Ab to AMA-1 were detected in babies experiencing a primary sub-microscopic infection. This result was not totally unexpected since among the 8 antigens studied, AMA1 was the most immunogenic with respect to Ab prevalence and levels. However, babies experiencing their first infection detected by microscopy made IgG to many of the antigens, including AMA1, MSP1-42, MSP2 (FC27), MSP3, RESA and LSAI (Table 5). The breadth of the response in babies appears to be similar to that reported for malaria-naïve adults who contacted malaria for the first time [27–33]. As expected, infants usually did not have symptomatic cases of *P. falciparum* until after the first 5 months of life and may have already experienced several previous asymptomatic infections. At the time of a clinical episode, most infants had IgG, and some were still producing IgM, to the 8 antigens.

The initial Ab responses to *P. falciparum* infections in both infants (Fig. 5, Additional file 1: Fig. S1) and malaria-naïve adults were short-lived [6, 29, 30, 32, 42]. However, the literature reports that secondary infections in adults resemble a secondary-type response that is significantly more robust, in that, higher titers [30, 35], increased number of Ab-secreting B cells [30], and functional Abs were found, e.g., Abs to MSP1 with growth inhibitory activity are produced [28]. In infants, the Ab response throughout the first year of life remains short-lived, with little or no evidence of increased levels of Abs being produced to any of the 8 antigens. Similar results have been reported previously. For example, Branch et al. published longitudinal data for MSP1-19 during the first year of life that was similar to those shown in Fig. 5 [42]. Keenhin et al. compared the Ab response of children and adults who were malaria-naïve prior to migrating into a malaria-endemic area [34]. They reported that the responses in children were slower to develop than those in adults. Overall, the slow maturation of anti-malarial IgG responses in infants is expected because Ab responses are typically of shorter duration, have a delayed onset, and differ in distribution of IgG isotypes than adults (reviewed in [44, 45]). It appears that during the first year of life babies begin to produce a rudimentary Ab IgG response, but the response does not reach maturity until later in life. If this is the case, then vaccinating infants during the first year of life for malaria may be effective, but for only for a short duration. Clearly, additional studies are needed to tease out the details of anti-malarial immunity in infants.

## Conclusions

In summary, the first infection in babies residing in malaria-endemic is almost always asymptomatic and self-resolving. Many of the first infections are sub-microscopic and may induce an IgM response to multiple *P. falciparum* antigens, but class-switching to IgG is restricted to more immunodominant antigens (e.g., AMA1). However, when primary infections were detected by microscopy, babies produced IgG to 6 of the 8 antigens studied. Therefore, both the IgM and IgG responses may occur before maternal IgG Abs wane completely. As reported previously, the primary Ab response is short-lived with both IgM and IgG Ab quickly declining over a few months. Subsequently, babies produce IgG responses upon re-infection, but the response is similar to that after primary infection. Even with repeated infections and clinical episodes, there was little evidence that infants produced a true secondary-type humoral response during the first year of life.

## Supplementary Information

**Additional file 1: Figure S1.** Comparison of antibody levels in babies at 1 week of life whose subsequent primary infections were sub-microscopic (n = 18) or detected by slide-microscopy (n = 39) Horizontal lines = medians. SUB = sub-microscopic infection; Slide = infection detected by microscopy.

**Additional file 2: Figure S2.** IgM antibody levels during the first year of life. Horizontal lines show the cut-off for antibody positivity. (n = 47 babies). A. AMA-1 (3D7), B. AMA1 (FVO); C. EBA-175; D. MSP2 (3D7); E. MSA2 (FC27) F. MSP1-42 (3D7); G. MSP1-42 (FVO); H. MSP3; I. RESA, J. LSA1, and K. CSP.

**Additional file 3: Figure S3.** Some infants had several IgG responses during the first year of life Note: MFI were similar between the primary and secondary IgG Ab response. Figure A-H show IgG responses of different infants (Baby#). Babies A-E had only 1 to 2 detected infections; whereas, babies F to H had multiple infections.

**Additional file 4: Figsure S4.** No correlation between antibody levels at birth and time to first infection. Ab levels (MFI) in plasma collected at the first visit (mean 9 ± 2 days) of each baby were plotted against time (days) to first infection that was detected by PCR or slide. Simple linear regression was used to determine if Ab levels to any of the antigens was associated with a significant delay in time to infection. No association was found (all R^2^ values were very low < 0.01 indicating no association) and all p values were non-significant (> 0.01), showing the regression lines did not differ significant from zero (horizontal). Babies who remained malaria-negative during the first year were assigned a value of 366 days as the time to first infection.

**Additional file 5: Figure S5.** No correlation between Ab levels at birth and number of infections detected by PCR. Antibody levels (MFI) for each baby at the first visit (mean 9 ± 2 days) were plotted against the number of times the baby was PCR-positive during the first year of life. No association between Ab levels at birth and number of PCR-detected infections was seen.

**Additional file 6: Figure S6.** No correlation between Ab levels at birth and number of infections detected by microscopy. Antibody levels (MFI) at the first visit (mean 9 ± 2 days) were plotted against the number of times each baby was slide-positive during the first year of life. No association between high Ab levels at birth and number of slide-positive infections was found.

**Additional file 7: Table S1.** Recombinant and synthetic antigens used in antibody assays.

## Data Availability

The dataset used/or analysed during the current study are available from the corresponding author on reasonable request.

## Abbreviations

Ab: Antibody
Abs: Antibodies
Ags: Antigens
AMA-1: Apical merozoite antigen-1
CSP: Circum-sporozoite protein
EBA-175: Erythrocyte binding antigen-175
IE: Infected erythrocyte
IgG: Immunoglobulin G
IgM: Immunoglobulin M
IVS: Intervillous space
LBW: Low birthweight
LSA-1: Liver-stage antigen-1
MFI: Median fluorescence intensity
MSP1-42: Merozoite surface protein 1—C-terminal 42 fragment
MSP2: Merozoite surface protein 2
MSP3: Merozoite surface protein 3
Pf: *Plasmodium falciparum*
PCR: Polymerase chain-reaction
PM: Placental malaria
PM +: Placental malaria-positive
PM-: Placental malaria-negative
RESA: Ring-stage erythrocyte surface antigen
3D7: Strains or isolates of *P. falciparum*
FVO: Strains or isolates of *P. falciparum*
FC27: Strains or isolates of *P. falciparum*
