# Supplementary material for: Characterization of the primary antibody response to Plasmodium falciparum antigens in infants living in a malaria-endemic area
Source: Malar J. 2022 Nov 19;21:346. doi: 10.1186/s12936-022-04360-x (PMC9675181; doi:10.1186/s12936-022-04360-x)
Supplement: Supplementary file 7 — Additional file 7: Table S1. Recombinant and synthetic antigens used in antibody assays. [file 12936_2022_4360_MOESM7_ESM.docx]

| Table S1. Recombinant and synthetic antigens used in antibody assays | | | | |
| --- | --- | --- | --- | --- |
| Antigen | Strain  or sequence | Type | Expression  system | Antigen amount used per million beads |
| MSP1_42_ | FVO | Recombinant | *Escherichia coli* | 1 µg |
| MSP1_42_ | 3D7 | Recombinant | *Escherichia coli* | 1 µg |
| MSP2 | FC27 | Recombinant | *Escherichia coli* | 1 µg |
| MSP2 | 3D7 | Recombinant | *Escherichia coli* | 1 µg |
| MSP3 | 3D7, C-Terminal | Recombinant | *Escherichia coli* | 0.2 µg |
| AMA1 | FVO | Recombinant | Yeast cells | 1 µg |
| AMA1 | 3D7 | Recombinant | Yeast cells | 1 µg |
| EBA175 | Region II | Recombinant | Yeast cells | 0.2 µg |
| RESA | [EENV]5-BSA | *Synthetic | n/a | 2.5 µg |
| CSP | [PNAN]5-BSA | *Synthetic | n/a | 2.5 µg |
| LSA1 | -BSA | *Synthetic | n/a | 4 nmol |
| * The peptide antigens were synthesized by AnaSpec. | | | | |
