# Supplementary material for: Characterization of the primary antibody response to Plasmodium falciparum antigens in infants living in a malaria-endemic area
Source: Malar J. 2022 Nov 19;21:346. doi: 10.1186/s12936-022-04360-x (PMC9675181; doi:10.1186/s12936-022-04360-x)
Supplement: Supplementary file 2 [file 12936_2022_4360_MOESM1_ESM.docx]

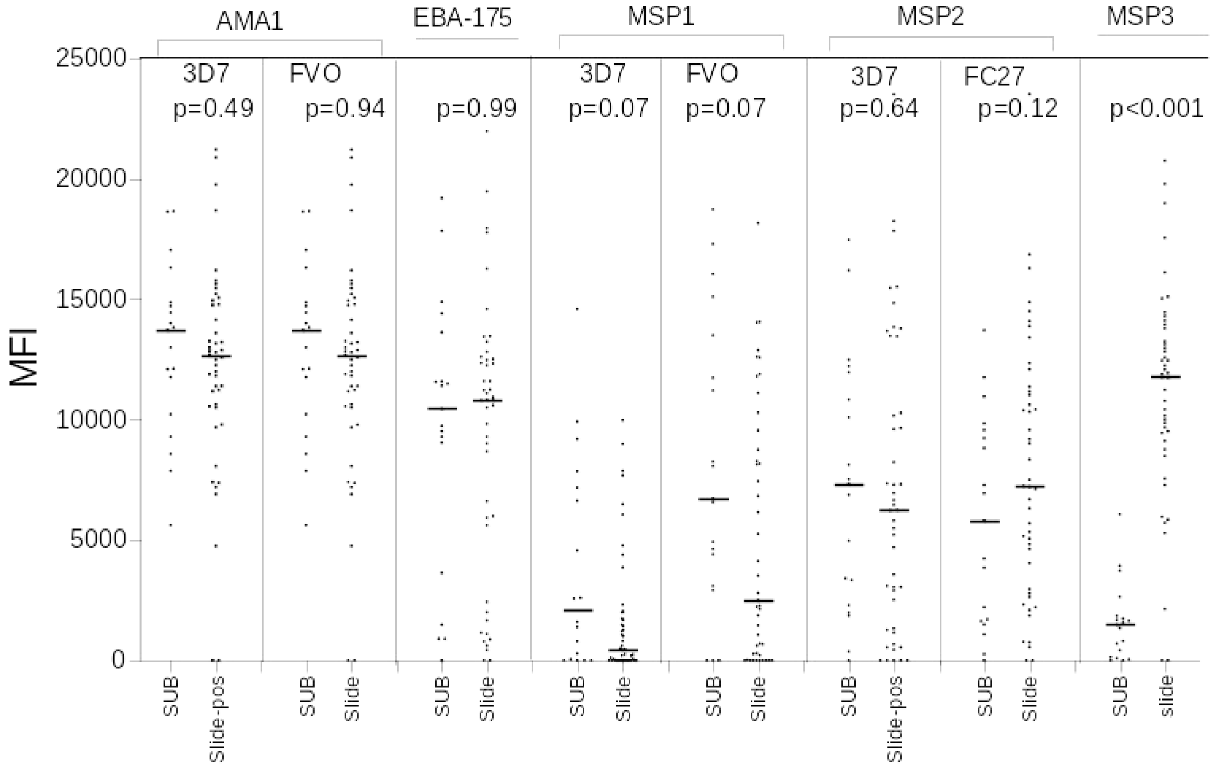

SUB = submicroscopic infection; Slide = infection detected by microscopy
