# Supplementary material for: Characterization of the primary antibody response to Plasmodium falciparum antigens in infants living in a malaria-endemic area
Source: Malar J. 2022 Nov 19;21:346. doi: 10.1186/s12936-022-04360-x (PMC9675181; doi:10.1186/s12936-022-04360-x)
Supplement: Supplementary file 3 [file 12936_2022_4360_MOESM2_ESM.docx]

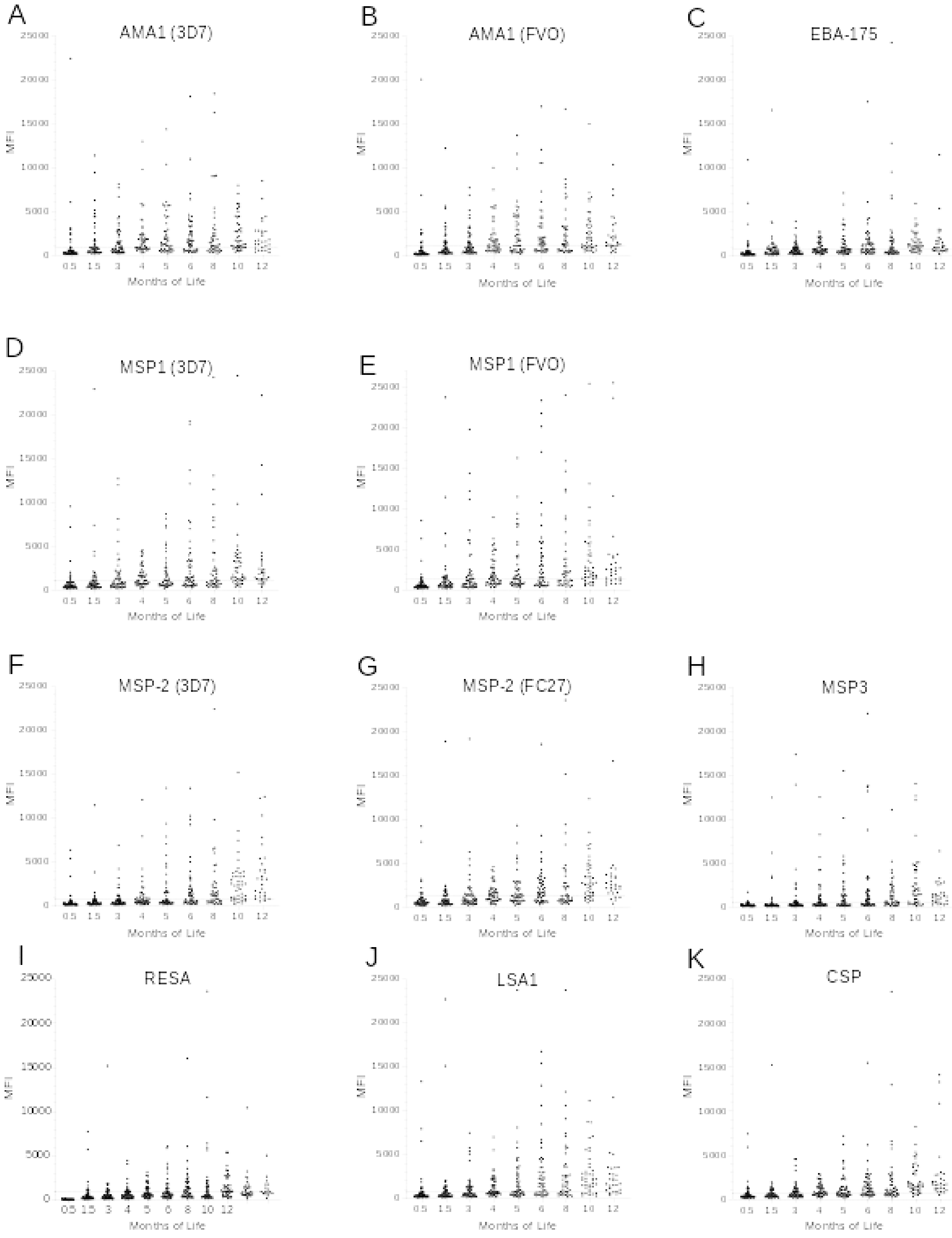
A
AMA1 (3D7)
B
AMA1 (FVO)
C
EBA-175
D
MSP1 (3D7)
E
MSP1 (FVO)
F
MSP-2 (3D7)
G
MSP-2 (FC27)
H
MSP3
I
RESA
J
LSA1
K
CSP
MFI
0
5000
10000
15000
20000
25000
Months of Life
0.5
1.5
3
4
5
6
8
10
12
