# Supplementary material for: Characterization of the primary antibody response to Plasmodium falciparum antigens in infants living in a malaria-endemic area
Source: Malar J. 2022 Nov 19;21:346. doi: 10.1186/s12936-022-04360-x (PMC9675181; doi:10.1186/s12936-022-04360-x)
Supplement: Supplementary file 4 [file 12936_2022_4360_MOESM3_ESM.docx]

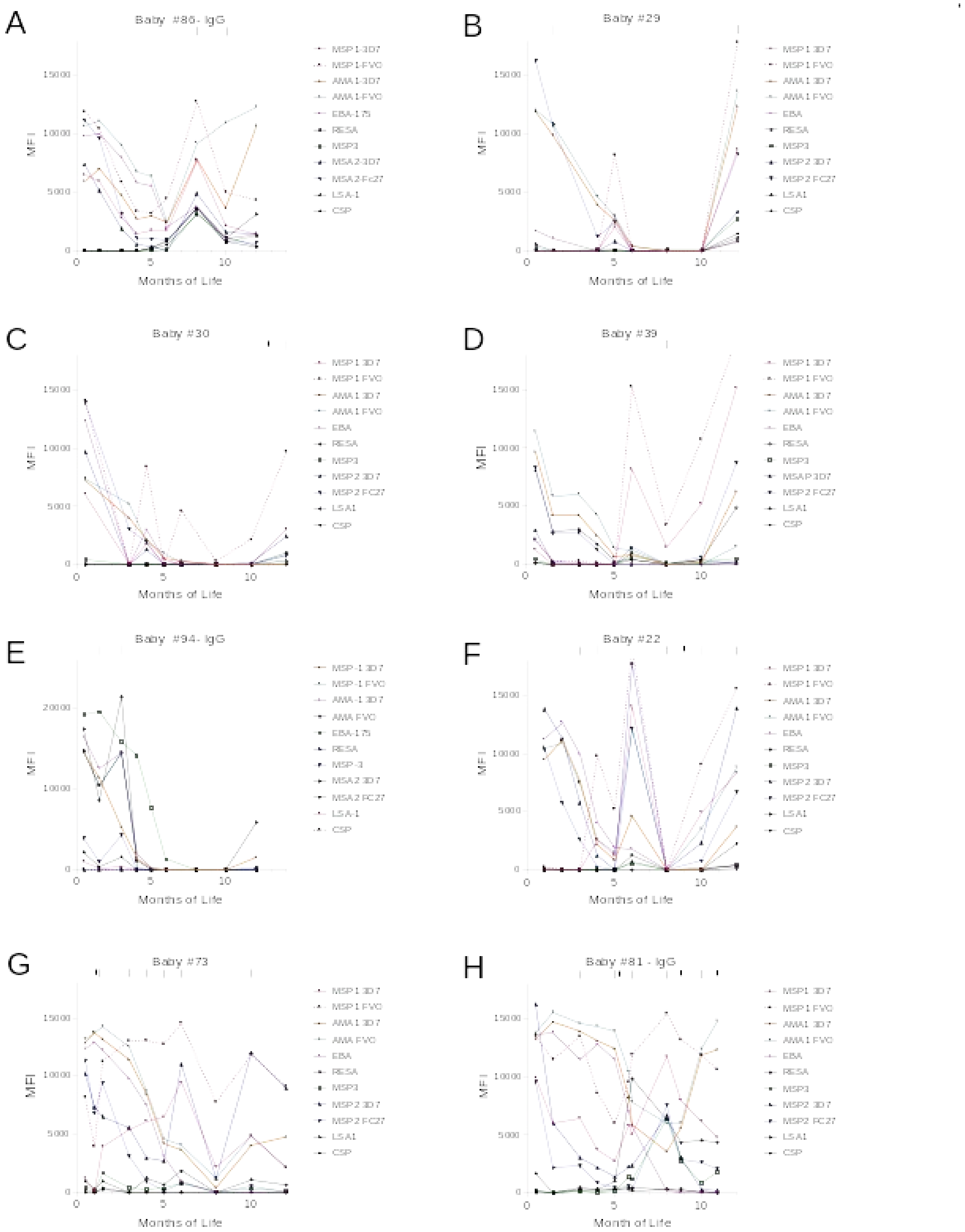
A
Baby #86- IgG
MFI
Months of Life
MSP1-3D7
MSP1-FVO
AMA1-3D7
AMA1-FVO
EBA-175
RESA
MSP3
MSA2-3D7
MSA2-Fc27
LSA-1
CSP
B
Baby #29
MFI
Months of Life
MSP1 3D7
MSP1 FVO
AMA1 3D7
AMA1 FVO
EBA
RESA
MSP3
MSP2 3D7
MSP2 FC27
LSA1
CSP
C
Baby #30
MFI
Months of Life
MSP1 3D7
MSP1 FVO
AMA1 3D7
AMA1 FVO
EBA
RESA
MSP3
MSP2 3D7
MSP2 FC27
LSA1
CSP
D
Baby #39
MFI
Months of Life
MSP1 3D7
MSP1 FVO
AMA1 3D7
AMA1 FVO
EBA
RESA
MSP3
MSAP 3D7
MSP2 FC27
LSA1
CSP
E
Baby #94- IgG
MFI
Months of Life
MSP-1 3D7
MSP-1 FVO
AMA-1 3D7
AMA FVO
EBA-175
RESA
MSP-3
MSA2 3D7
MSA2 FC27
LSA-1
CSP
F
Baby #22
MFI
Months of Life
MSP1 3D7
MSP1 FVO
AMA1 3D7
AMA1 FVO
EBA
RESA
MSP3
MSP2 3D7
MSP2 FC27
LSA1
CSP
G
Baby #73
MFI
Months of Life
MSP1 3D7
MSP1 FVO
AMA1 3D7
AMA FVO
EBA
RESA
MSP3
MSP2 3D7
MSP2 FC27
LSA1
CSP
H
Baby #81 - IgG
MFI
Month of Life
MSP1 3D7
MSP1 FVO
AMA1 3D7
AMA1 FVO
EBA
RESA
MSP3
MSP2 3D7
MSP2 FC27
LSA1
CSP
