# Supplementary material for: Characterization of the primary antibody response to Plasmodium falciparum antigens in infants living in a malaria-endemic area
Source: Malar J. 2022 Nov 19;21:346. doi: 10.1186/s12936-022-04360-x (PMC9675181; doi:10.1186/s12936-022-04360-x)
Supplement: Supplementary file 5 [file 12936_2022_4360_MOESM4_ESM.docx]

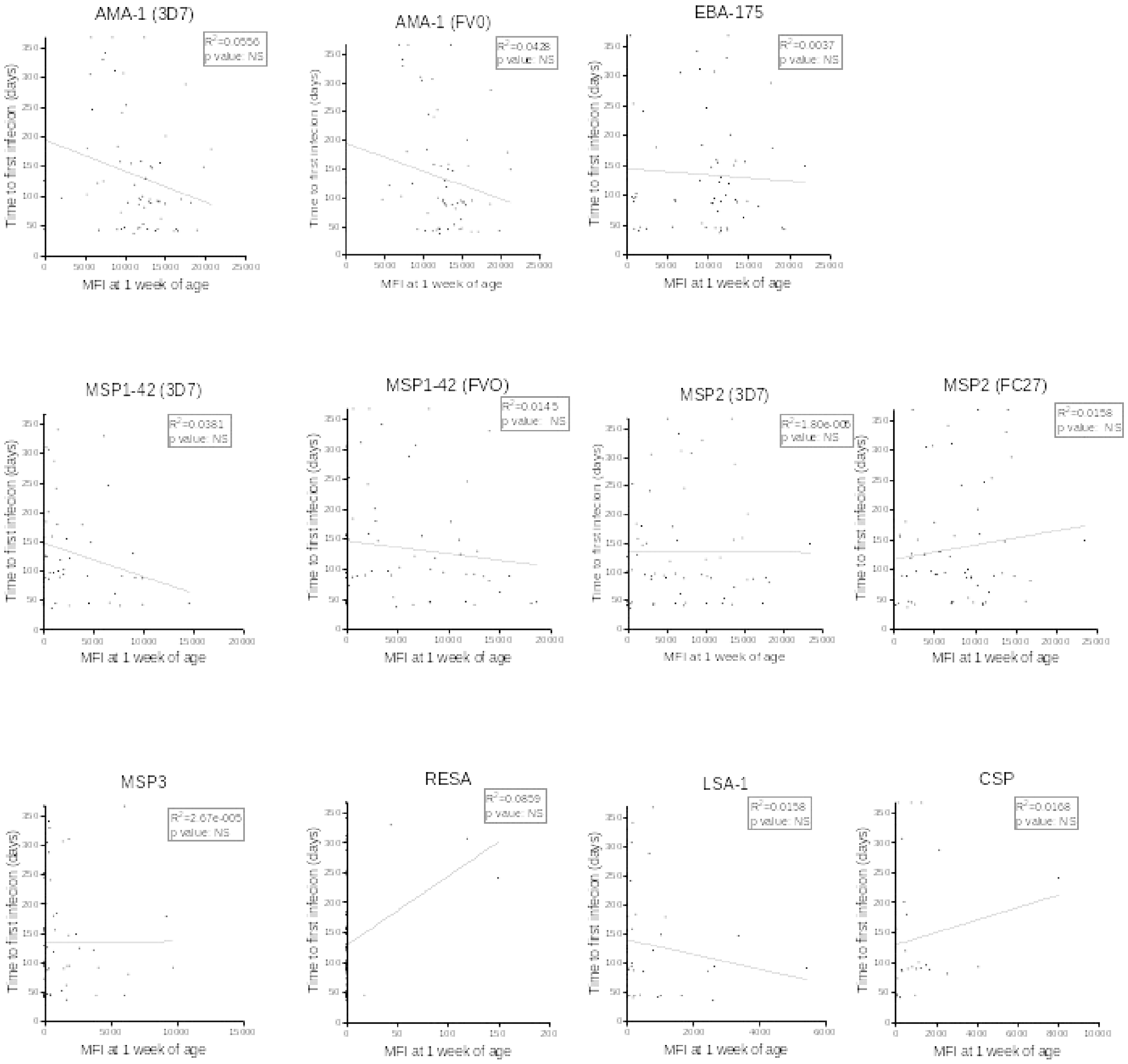
AMA-1 (3D7)
R2=0.0556
p value: NS
AMA-1 (FV0)
R2=0.0428
p value: NS
EBA-175
R2=0.0037
p value: NS
MSP1-42 (3D7)
R2=0.0381
p value: NS
MSP1-42 (FVO)
R2=0.0145
p value: NS
MSP2 (3D7)
R2=1.80e-005
p value: NS
MSP2 (FC27)
R2=0.0158
p value: NS
MSP3
R2=2.67e-005
p value: NS
RESA
R2=0.0859
p value: NS
LSA-1
R2=0.0158
p value: NS
CSP
R2=0.0108
p value: NS
Time to first infecion (days)
MFI at 1 week of age
