# Supplementary material for: Characterization of the primary antibody response to Plasmodium falciparum antigens in infants living in a malaria-endemic area
Source: Malar J. 2022 Nov 19;21:346. doi: 10.1186/s12936-022-04360-x (PMC9675181; doi:10.1186/s12936-022-04360-x)
Supplement: Supplementary file 6 [file 12936_2022_4360_MOESM5_ESM.docx]

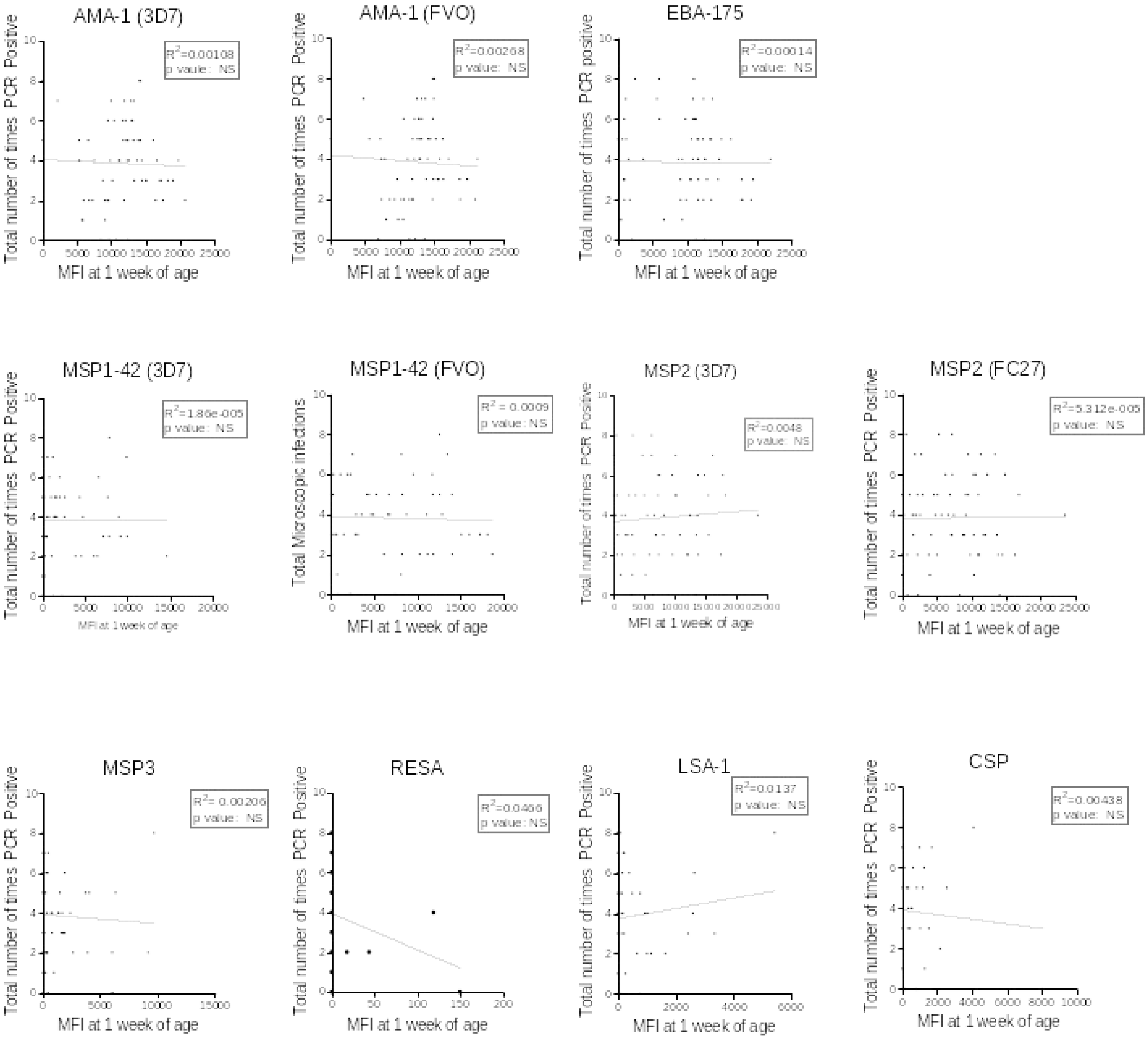

AMA-1 (3D7)
R2=0.00108
p value: NS
AMA-1 (FVO)
R2=0.00268
p value: NS
EBA-175
R2=0.00014
p value: NS
MSP1-42 (3D7)
R2=1.86e-005
p value: NS
MSP1-42 (FVO)
R2 = 0.0009
p value: NS
MSP2 (3D7)
R2=0.0048
p value: NS
MSP2 (FC27)
R2=5.312e-005
p value: NS
MSP3
R2= 0.00206
p value: NS
RESA
R2=0.0466
p value: NS
LSA-1
R2=0.0137
p value: NS
CSP
R2=0.00438
p value: NS
Total number of times PCR Positive
Total Microscopic infections
MFI at 1 week of age
