# Supplementary material for: Characterization of the primary antibody response to Plasmodium falciparum antigens in infants living in a malaria-endemic area
Source: Malar J. 2022 Nov 19;21:346. doi: 10.1186/s12936-022-04360-x (PMC9675181; doi:10.1186/s12936-022-04360-x)
Supplement: Supplementary file 7 [file 12936_2022_4360_MOESM6_ESM.docx]

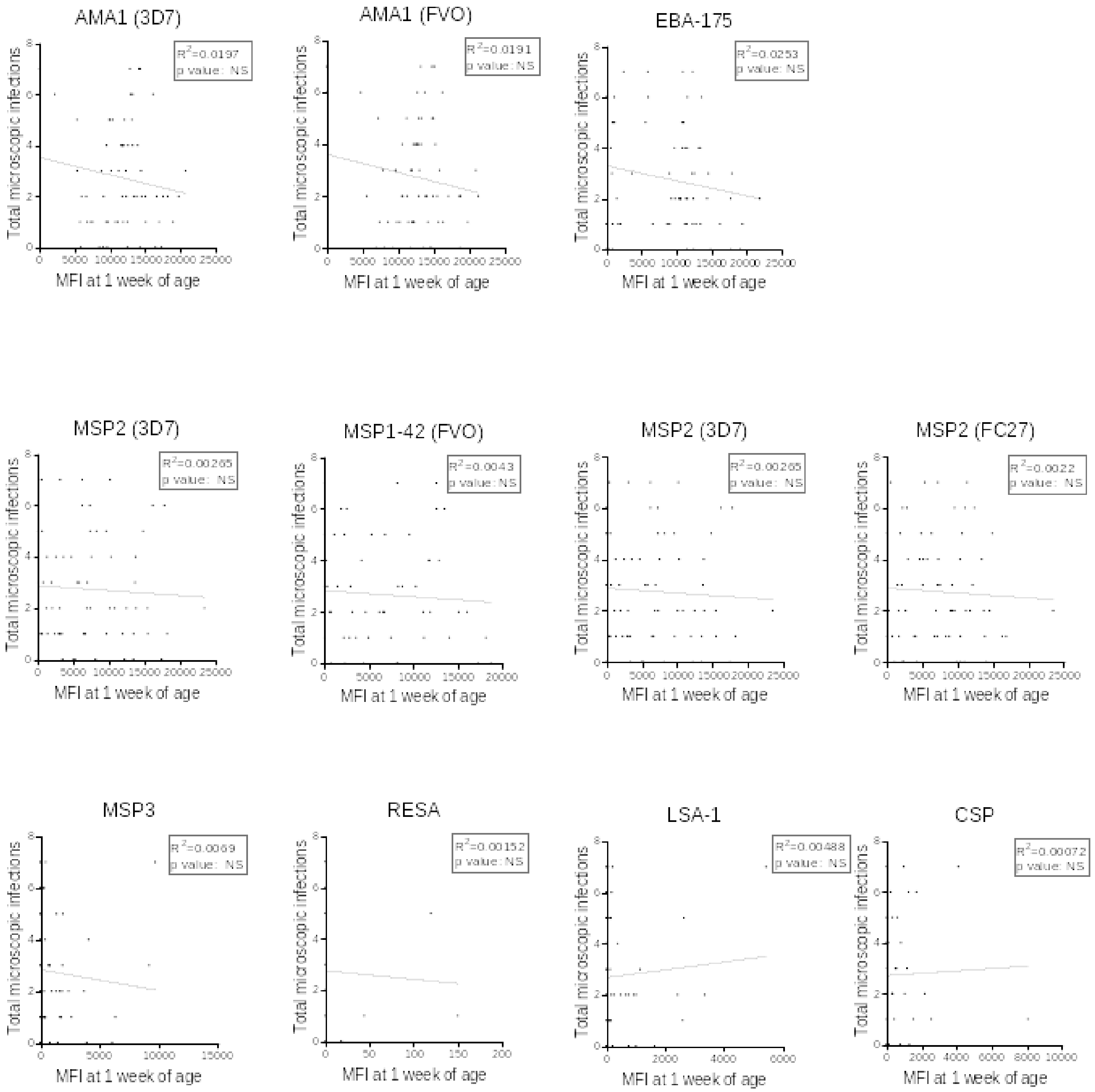
AMA1 (3D7)
R2=0.0197
p value: NS
AMA1 (FVO)
R2=0.0191
p value: NS
EBA-175
R2=0.0253
p value: NS
MSP2 (3D7)
R2=0.00265
p value: NS
MSP1-42 (FVO)
R2=0.0043
p value: NS
MSP2 (3D7)
R2=0.00265
p value: NS
MSP2 (FC27)
R2=0.0022
p value: NS
MSP3
R2=0.0069
p value: NS
RESA
R2=0.00152
p value: NS
LSA-1
R2=0.00488
p value: NS
CSP
R2=0.00072
p value: NS
Total microscopic infections
MFI at 1 week of age
